# Changes in employment status and income before and after newly diagnosed depressive disorders in Taiwan: a matched cohort study using controlled interrupted time series analysis

**DOI:** 10.1017/S2045796023000562

**Published:** 2023-06-30

**Authors:** Yu-Ling Chen, Wei-Hsiang Liao, Shih-Heng Wang, Yin-Ju Lien, Chia-Ming Chang, Shih-Cheng Liao, Wei-Lieh Huang, Chi-Shin Wu

**Affiliations:** 1 National Center for Geriatrics and Welfare Research, National Health Research Institutes, Zhunan, Taiwan; 2 Department of Physical Education, National Taiwan University of Sport, Taichung City, Taiwan; 3 Department of Psychiatry, National Taiwan University Hospital, Taipei, Taiwan; 4 Department of Public Health, College of Public Health, China Medical University, Taichung, Taiwan; 5 Department of Health Promotion and Health Education, National Taiwan Normal University, Taipei, Taiwan; 6 Department of Psychiatry, Chang Gung Memorial Hospital at Linkou, Tao-Yuan, Taiwan; 7 Department of Psychiatry, College of Medicine, National Taiwan University, Taipei, Taiwan; 8 Department of Psychiatry, National Taiwan University Hospital, Hsin-Chu Branch, Hsinchu, Taiwan; 9 Department of Psychiatry, National Taiwan University Hospital, Yunlin Branch, Douliu, Taiwan

**Keywords:** depression, employment, income, interrupted time series analysis, propensity score

## Abstract

**Aims:**

We explored long-term employment status and income before and after depression diagnosis among men and women and at different working ages in Taiwan.

**Methods:**

Data from 2006 to 2019 were obtained from the National Health Insurance Research Database (NHIRD). Individuals with newly diagnosed depressive disorder aged 15 to 64 years during the study period were identified. An equal number of individuals without depression were matched for their demographic and clinical characteristics. Employment outcomes included employment status, which was categorized into employed or unemployed, and annual income. Based on the occupation categories and monthly insurance salary recorded in the Registry for Beneficiaries of the NHIRD, a subject was defined as unemployed if he or she differed from the income earner or the occupation category was unemployed. Monthly income was defined as zero for unemployed subjects and proxied as monthly insurance salary for others. Annual income was the sum of monthly income in each observation year.

**Results:**

A total of 420,935 individuals with depressive disorder were included in the study, and an equal number of individuals with not diagnosed depression served as controls. Employment rate and income were lower in the depression group than in the control group before the year of diagnosis, with a difference of 5.7% in employment rate and USD 1,173 in annual income. This gap increased considerably after the year of diagnosis (7.3% in employment rate and USD 1,573 in annual incomes) and further widened in the subsequent years (8.1% in employment rate and USD 2,006 in annual incomes in the 5th following year). The drops in the employment rate and income caused by depression were more evident in men and older age groups than in women and younger age groups, respectively. However, the reduction in employment rate and income in the following years after the diagnosis was more considerable among younger age groups.

**Conclusions:**

The effect of depression on employment status and income was significant during the year of diagnosis and continued afterwards. The effect on employment outcomes varied between genders and across all age groups.

## Introduction

Depressive disorders are among the most burdensome illnesses worldwide (GBD 2019 Diseases and Injuries Collaborators, 2020). The overall cost of depression in the United States was estimated at USD 326.2 billion in 2020, with 73.2% of the costs attributed to workplace-related costs (Greenberg *et al.*, 2021). Several studies have demonstrated that patients with depressive disorders have a higher risk of subsequent unemployment or low income (Campbell *et al.*, 2022; Dooley *et al.*, 2000; Jefferis *et al.*, 2011; Luo *et al.*, 2010; Whooley *et al.*, 2002). Additionally, the association between depression and employment status changes may be bidirectional (Dooley *et al.*, 2000; Jefferis *et al.*, 2011). Unemployment or inadequate employment could increase the likelihood of having depressive symptoms (Dooley *et al.*, 2000; Jefferis *et al.*, 2011; Yoo *et al.*, 2016). Furthermore, the disease course of depression can be chronic or recurrent, leading to long-term effects on employment outcomes (Dobson *et al.*, 2021; Hakulinen *et al.*, 2021; Mojtabai *et al.*, 2015).

Several factors may modify the association between depression and employment outcomes. Studies have shown that the impact of depressive disorders on yearly earnings is more prominent among men than among women (Hakulinen *et al.*, 2021; Luo *et al.*, 2010). Additionally, the effects of depression may vary across age groups. Some studies have shown that older adults are more likely to reduce their work participation due to depression (Dewa *et al.*, 2002; Rytsälä *et al.*, 2007). However, there may be no association between adolescent depression and subsequent employment or income in adulthood (Callander, 2016; Naicker *et al.*, 2013). The severity of depressive disorder also influences employment outcomes, such as job-seeking and sickness absence (Hakulinen *et al.*, 2021; Luo *et al.*, 2010).

Existing evidence that demonstrates the effect of depression on employment status and income are primarily from Western studies (Berndt *et al.*, 2000; Callander, 2016; Dewa *et al.*, 2002; Dobson *et al.*, 2022; Dooley *et al.*, 2000; Hakulinen *et al.*, 2021, 2016; Jefferis *et al.*, 2011; Luo *et al.*, 2010; Rytsälä *et al.*, 2007). In Taiwan, a high-income Asian country, the prevalence of depressive disorders was relatively low (Liao *et al.*, 2012); however, it raised from 1.61% in 2007 to 1.92% in 2016, and this trend was seen across all age groups, both genders, different income levels, and employment status (Wang *et al.*, 2022). The impact of depressive disorders on economic burden raised public concerns. In addition, the long-term effect of depressive disorder on employment outcomes was not yet fully explored. Hence, the current study applied a population-representative sample over 10-year follow-up periods to explore the association between depression and employment status and income among different genders and age groups in Taiwan, and the possible effects of depression severity on employment outcomes were also examined. We hypothesized that depression would be associated with a reduced employment rate and income. In addition, the association would vary across age groups, genders, and the severity of the depression. The findings of this study are anticipated to help bridge the limited understanding of the impact of depressive disorders on employment outcomes in Taiwan and inform the mental healthcare policy for caring for the workforce.

## Methods

### Data source

We conducted a matched cohort study using controlled interrupted time series analyses of the effect of depressive disorders on employment outcomes. Data were obtained from the National Health Insurance Research Database (NHIRD), maintained by Taiwan’s National Health Insurance Program. This is a universal compulsory program that includes approximately 99% of the 23 million Taiwanese population. The NHIRD provided data on insured clinical diagnoses, treatments, investigation items and medical and surgical procedures. The clinical diagnoses were coded based on the International Classification of Diseases (ICD)-9 before 2016 and updated to ICD-10 since 2016. The accuracy of major psychiatric disorders, including depressive disorders, has been documented (Wu *et al.*, 2020). The Registry for National Health Insurance (NHI) beneficiaries included basic demographic information of the insured individual, such as birth year, monthly salary income, and the identification of income earners. Sex was also extracted from the Registry database and will be used in the study to conceptualize gender. Identification is encrypted to protect personal privacy. This study was approved by the Research Ethics Committee of the National Health Research Institute (EC1101103-E).

### Study population

This study included 2006–2019 NHIRD data from all insured individuals, except for military conscripts and inmates in correctional facilities, totalling approximately 23 million (Lin *et al.*, 2018). Patients with depressive disorders were identified based on ICD codes, including major depressive disorder (ICD-9-CM:296.2, 296. and; ICD-10: F32.0-F32.9, F33.0-F33.9) and minor depression (ICD-9-CM:300.4, 311 and ICD-10-CM: F34.1). The accuracy of the diagnostic code for major depressive disorders was good (the sensitivity and positive predictive values were both 0.86), and the accuracy for minor depression was fair (the sensitivity and positive predictive values were 0.64 and 0.61, respectively) (Wu *et al.*, 2020). In order to have a 5-year observation period before and after the year of diagnosis, we only included those who had newly diagnosed depressive disorders between 2011 and 2014. A total of 995,998 patients with depressive disorders from 2011 to 2014 were identified. The date of the first diagnosis of depressive disorder was defined as the index date. After excluding prevalent patients whose index date was before 2011 (*n* = 406,830), 589,168 newly diagnosed cases were included. Cases with missing data on gender, year of birth, home city, or employment status were excluded (*n* = 26,937). We further excluded 120,035 subjects who were not of working age and were younger than 15 years or older than 64 years. Patients diagnosed with schizophrenia or bipolar disorder before the index date were excluded (*n* = 21,261) because these psychiatric disorders are severe and highly impact occupational function. Finally, 420,935 patients with newly diagnosed depressive disorders were included.

For each newly diagnosed patient with depressive disorder, we identified potential comparisons who matched them by birth year and gender from the Registry for NHI beneficiaries. Then we excluded those having missing data on demographic variables or any diagnosis of depressive disorders, schizophrenia, or bipolar disorder before the index date of the cases. Given that the sample size of cases with depressive disorders was sufficiently large, we only selected one comparison using simple random sampling. The index date of the matched comparison was the same as that of the corresponding case. Eventually, the same number of comparisons was included.

The index year was defined as 6 months before and 6 months after the index date. Subjects with depressive disorders and comparisons were observed for 5 years before and 5 years after the index year (Supplementary Figure S1).

### Employment outcomes

Employment outcomes included employment status and income. The Registry for NHI beneficiaries included occupation categories, monthly insurance salary, and the encrypted identification of insured subjects and income earners. The Registry for NHI beneficiaries is updated monthly. The subject was defined as unemployed if the identification of the insured person was different from that of the income earner or if the occupation category was unemployment. For each year, employment status was categorized as unemployment if the unemployed months were equal to or more than 6 months. To determine the employment rate for each year analysed, the count of employed individuals was divided by the total number of subjects included in the study for that specific year.

If the subjects were unemployed in a month, the monthly income was defined as zero; otherwise, the monthly income was proxied as the monthly insurance salary. Annual income was the sum of the monthly income in each observation year. Annual income in New Taiwan Dollar (NTD) was converted to USD based on a 30.9 to 1 exchange rate between NTD and USD in 2019.

### Baseline demographic and clinical characteristics

The demographic and clinical characteristics of the cases and comparisons in the index year included age group, gender, urbanicity and general physical health. Urbanicity was determined by the results of cluster analysis using five variables: population density, physician density, population ratio of people with a college degree or more, older adults aged 65 or above and agriculture workers (Liu *et al.*, 2006). General physical health was measured using the Charlson comorbidity index (CCI) score (Glasheen *et al.*, 2019), which is the sum of the weighted scores of the 19 clinical diagnoses, including myocardial infarction, congestive heart failure, peripheral vascular disease, cerebrovascular disease, chronic obstructive pulmonary disease, dementia, paraplegia and hemiplegia, diabetes, diabetes with complications, renal disease, mild liver disease, moderate or severe liver disease, peptic ulcers, rheumatic disease, acquired immunodeficiency syndrome, cancer, leukaemia, lymphoma and metastatic solid tumour. Three categories were created according to the CCI scores: 0, 1, 2 and ≥3.

### Statistical analysis

Descriptive statistics of the cases and comparisons were reported, and the average employment rate and annual income of the cases and comparisons for the observation periods were plotted.

Given that the baseline demographic and clinical characteristics of the cases and comparisons were different, we used propensity-score matching to eliminate these differences. We used greedy nearest neighbour matching and selected the comparison whose propensity score best matches the propensity score of each case. The age and gender were hard matched on. The matching process is done sequentially and without replacement. To ensure accurate matching, we set a caliper requirement of 0.5, meaning that pairs from the two groups are matched only if the difference in their propensity score logits is less than or equal to 0.5 times the common standard deviation of the propensity score logits. The 0.1 threshold of the standardized mean difference was used to assess whether the balance was achieved by matching (Austin, 2009).

The impact of depression may have been initiated before the index date or delayed by several months. The effects on employment outcome were not fully stabilized in the transition period; therefore, we chose not to include the index year in our analysis. Each subject underwent 10 repeated measures, 5 years before and after the index year. We used segmented regression analysis of a controlled interrupted time series design to assess the longitudinal effect of depression on employment status and annual income (Lopez Bernal *et al.*, 2018). The model was fitted to the following equation:

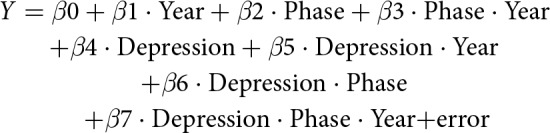


*Y* is the employment status or the annual income; Year is the time point of the data (0 is the index year, 1 is the first year after the index year, and −2 is the 2 years preceding the index year); Phase indicates ‘before/after the index year’ (0 before the index year and 1 after the index year); and Depression indicates depressive disorders (0 for controls or 1 for subjects with depressive disorders). In this model, *β*0–*β*3 estimate the parameters of interest among the controls. *β*0 estimates the odds of being employed and baseline annual income in the index year. *β*1 estimates the trend in employment outcomes before the index year. *β*2 estimates the change in employment outcomes immediately after the index year. *β*3 estimates the change in trends after the index year. *β*4–*β*7 estimate the differences of the case groups with the comparison groups. *β*4 estimates the difference in the odds of being employed or annual income in the index year between a case with depression and a control without depression. *β*5 estimates the difference in the trends before the index year. *β*6 estimates the level change in employment outcomes during the index year. Finally, *β*7 estimates the difference in the change in trend after the index year.

We used a generalized estimating equation (GEE) model for employment status (binary outcome) with a logit link to compute the adjusted odds ratios (ORs) and 95% confidence intervals (CIs)(Hanley *et al.*, 2003). For annual income, we used a GEE model with an identity link function to calculate the coefficients and 95% CIs. A first-order autoregressive correlation structure was used for both models. In subgroup analyses, we divided subjects based on different age groups (15–24, 25–34, 35–44, 45–54 and 55–64) and genders. Distinct models were conducted for these subgroups. In addition, we assessed the effect of severity of depression based on the subtype of depressive disorder (major vs. minor depression) and psychiatric hospitalization in the index year (‘yes’ or ‘no’) among cases. A sensitivity analysis was conducted using the sample before propensity-score matching. The differences in Charlson comorbid index score were adjusted in regression models. All statistical analyses were performed using SAS (version 9.4; SAS Institute, Inc., Cary, NC, USA). Statistical significance was assessed using 95% CIs or a *p* value < 0.05.

## Results

Table 1 shows the baseline characteristics of the study sample before and after propensity-score matching. All the cases included in the study experienced either major (*n* = 329,316) or minor depression (*n* = 91,619). Before matching, among the newly diagnosed patients with depressive disorders, 62.5% were women. Overall, the majority were over 25 years of age, and more than half lived in the urban area; however, patients with depressive disorders had higher CCI scores than the controls. Among the cases, 78.2% were diagnosed with major depressive disorders (Supplementary Table S1) and 1.3% were hospitalized in a psychiatric ward in the index year (Supplementary Table S2). The incident cases of depressive disorder declined from 111,180 in 2011 to 100,705 in 2014. After propensity-score matching, 56,972 (13.5%) cases and an equal number of controls were excluded from the analysis. The standardized mean differences of all variables were <0.1.

**Table 1.** Sample characteristics before and after matching

	Before matches			After matched		
	Control, *n* (%)	Case, *n* (%)	STDDIF	Control, *n* (%)	Case, *n* (%)	STDDIF
Age category						
15−24	46,985 (11.2)	46,985 (11.2)	0.00	44,278 (12.2)	44,278 (12.2)	0.00
25−34	80,126 (19.0)	80,126 (19.0)	0.00	74,237 (20.4)	74,237 (20.4)	0.00
35−44	93,327 (22.2)	93,327 (22.2)	0.00	81,648 (22.4)	81,648 (22.4)	0.00
45−54	104,922 (24.9)	104,922 (24.9)	0.00	86,750 (23.8)	86,750 (23.8)	0.00
55−64	95,575 (22.7)	95,575 (22.7)	0.00	77,050 (21.2)	77,050 (21.2)	0.00
Gender						
Men	157,936 (37.5)	157,936 (37.5)	0.00	131,509 (36.1)	131,509 (36.1)	0.00
Women	262,999 (62.5)	262,999 (62.5)	0.00	232,454 (63.9)	232,454 (63.9)	0.00
Index year						
2011	111,180 (26.4)	111,180 (26.4)	0.00	96,108 (26.4)	97,666 (26.8)	−0.02
2012	106,714 (25.4)	106,714 (25.4)	0.00	91,937 (25.3)	90,507 (24.9)	0.01
2013	102,336 (24.3)	102,336 (24.3)	0.00	88,183 (24.2)	90,528 (24.9)	−0.02
2014	100,705 (23.9)	100,705 (23.9)	0.00	87,735 (24.1)	85,262 (23.4)	0.02
Residential area						
Urban	221,391 (52.6)	222,102 (52.8)	0.00	194,462 (53.4)	191,288 (52.6)	0.02
Suburban	156,514 (37.2)	155,682 (37.0)	0.00	133,824 (36.8)	135,432 (37.2)	−0.01
Rural	43,030 (10.2)	43,151 (10.3)	0.00	35,677 (9.8)	37,243 (10.2)	−0.01
CCI score						
0	355,547 (84.5)	299,166 (71.1)	0.33	298,845 (82.1)	298,845 (82.1)	0.00
1′	41,444 (9.8)	68,716 (16.3)	−0.19	41,290 (11.3)	40,827 (11.2)	0.00
2′	14,632 (3.5)	27,786 (6.6)	−0.14	14,574 (4.0)	14,979 (4.1)	−0.01
3 or more	9,312 (2.2)	25,267 (6.0)	−0.19	9,254 (2.5)	9,312 (2.6)	0.00

STDDIF, standardized difference; index year, year of depression diagnosis; CCI, Charlson comorbidity index.

**Figure 1.**
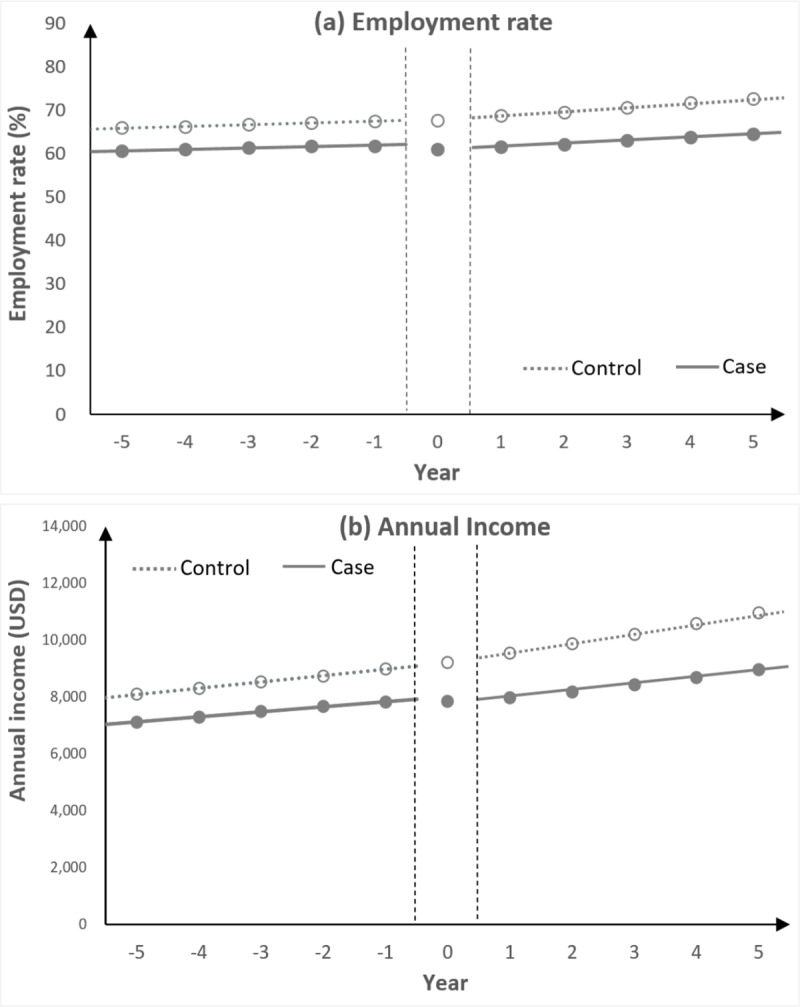
Employment rate (a) and annual income (b) in case and control groups before and after the index year.

Figure 1 and Supplementary Table S3 illustrate the level changes and trends of the average employment rate (a) and annual income (b) among the depression and comparison groups. In the year before the index year, the employment rates (61.9%) and annual incomes (USD 7,813) in the depression group were lower than those (67.6% and USD 8,986, respectively) in the comparison group. After the index year, employment rate and annual incomes were 61.6% and USD 7,891 for the depression groups and 68.8% and USD 9,554 for the comparison group, respectively. The differences increased immediately after the index year (from 5.7% to 7.3% in employment rate and from USD 1,173 to USD 1,573 in annual incomes) and further widened in subsequent years (8.1% in employment rate and USD 2,006 in annual incomes in the 5th year after the index year).

Table 2 shows the estimates using GEE model. Compared with the control group, the level change in the employment rate was 3.8% less (ORs = 0.962 [0.954–0.970]). The results also showed that the difference in the trend change after the index year of the employment rate was 0.4% (ORs = 0.996 [0.992–0.999]) less for the cases with depression. Similar patterns of average annual income were observed between the two groups. Compared to the control group, the level change was USD 227 (202–251) decrease, and the difference in the trend change after the index year was USD 41 (31–52) decrease per year in the following years. The results in sensitivity analyses using sample before matching were generally consistent, although the coefficient was slightly different (Supplementary Table S4).

**Table 2.** Overall controlled interrupted time series analysis, using generalized estimating equations with autoregressive correlation structure

	Employment status		Annual income	
Variables	Odds ratios (95% CI)	*p* value	Coefficient (95% CI)	*p* value
Intercept (*β*0)	2.168 (2.151, 2.185)	<0.001	9224 (9193, 9256)	<0.001
Year (*β*1)	1.043 (1.041, 1.045)	<0.001	316 (310, 321)	<0.001
Phase (*β*2)	0.992 (0.986, 0.997)	0.004	6 (−11, 23)	0.494
Phase × Year (*β*3)	0.984 (0.982, 0.987)	<0.001	−61 (−68, −53)	<0.001
Depression (*β*4)	0.772 (0.764, 0.781)	<0.001	−1230 (−1273, −1187)	<0.001
Depression × Year (*β*5)	0.992 (0.990, 0.995)	<0.001	−52 (−59, −45)	<0.001
Depression × Phase (*β*6)	0.962 (0.954, 0.970)	<0.001	−227 (−251, −202)	<0.001
Depression × Phase × Year (*β*7)	0.996 (0.992, 0.999)	0.012	−41 (−52, −31)	<0.001

CI, confidence interval; *β*1, Trends in the control groups; *β*2, Level change in the index year for the control group; *β*3, Trend change after the index year for the control groups; *β*4, Difference in intercept in index year between the case and control groups; *β*5, Trend differences between case and control groups; *β*6, Difference in level change in the index year between the case and control groups; *β*7, Difference in trend change after the index year between the case and control groups

### Subgroup analysis of the level change of employment outcomes

Table 3 shows that the decrease in the employment rate among men was greater than that among women (ORs = 0.950 [0.937–0.963] for men; 0.969 [0.959–0.979] for women; *p* value = 0.014). The reduction in annual income was also more significant among men (USD 320 [275–366]) than women (USD 173 [145–202]).

**Table 3.** Subgroups and stratified analysis on the differences in level change in the index year between case and control groups

	Employment status		Annual income	
Groups	Odds ratios (95% CI)	*p* value	Coefficient (95% CI)	*p* value
Gender				
Women	0.969 (0.959, 0.979)	<0.001	−173 (−202, −145)	<0.001
Men	0.950 (0.937, 0.963)	<0.001	−320 (−366, −275)	<0.001
Age groups, years				
15−24	0.989 (0.945, 1.035)	0.631	−63 (−121, −5)	0.034
25−34	0.968 (0.943, 0.993)	0.014	−185 (−240, −131)	<0.001
35−44	0.940 (0.922, 0.958)	<0.001	−302 (−351, −252)	<0.001
45−54	0.966 (0.950, 0.981)	<0.001	−188 (−236, −139)	<0.001
55−64	0.938 (0.923, 0.953)	<0.001	−338 (−400, −277)	<0.001
Subgroups of depression patients, by severity				
Major vs. minor	0.974 (0.958, 0.990)	0.002	−148 (−199, −97)	<0.001
Hospitalized vs non-hospitalized	0.874 (0.814, 0.939)	<0.001	−593 (−793, −392)	<0.001

CI, confidence interval

Two-by-two comparisons using Z tests

Employment status: women vs. men (*p* < 0.001); age 25–34 vs. age 35–44 (*p* = 0.004); age 35–44 vs. age 45–54 (*p* < 0.001); age 45–54 vs. age 55–64 (*p* < 0.001); and age 25–34 vs. age 55–64 (*p* = 0.004); other comparisons were not significant.

Annual income: women vs. men (*p* < 0.001); age 15–24 vs. age 25–34 (*p* < 0.001); age 15–24 vs. age 35–44 (*p* < 0.001); age 15–24 vs. age 45–54 (*p* < 0.001); age 15–24 vs. age 55–64 (*p* < 0.001); age 25–34 vs. age 35–44 (*p* < 0.001); age 25–34 vs. age 55–64 (*p* < 0.001); age 35–44 vs. age 45–54 (*p* < 0.001); and age 45–54 vs. age 55–64 (*p* < 0.001); other comparisons were not significant.

Patients aged 55–64 years had the most considerable reduction in employment rates (ORs = 0.938 [0.923–0.953]) and income (USD 338 [277–400]). Patients aged 15–24 had the least reduction of USD 63 (5–121) of their annual income and no significant decrease in the employment rate. Among patients with depressive disorders, those with major depression or psychiatric hospitalization in the index year had a greater reduction in employment rate and annual income than those with minor depression or without psychiatric hospitalization, respectively.

### Subgroup analysis of the difference in the change in the trend of employment outcomes

Table 4 shows that women with depressive disorders had a 0.5% decrease in the trend in the employment rate (ORs = 0.995 [0.991–0.999]), which indicates that the gap further increased after the index year. However, there was no significant trend change among men. In terms of age groups, the change in trend in the employment rate decreased markedly among those aged 15–24 years. In contrast, the trend change increased among those aged 25–34 and 35–44, which indicated the gap in employment rate between depression and control groups reduced gradually after the index year. There is no significant difference in the change in trend among those aged 45–54 and 55–64.

**Table 4.** Subgroups and stratified analysis on the differences of trends and changes 5 years after the index year between case and control groups

	Employment status		Annual income	
Groups	Odds ratios (95% CI)	*p* value	Coefficient (95% CI)	*p* value
Gender				
Women	0.995 (0.991, 0.999)	0.011	−44 (−57, −32)	<0.001
Men	0.997 (0.992, 1.003)	0.374	−37 (−56, −17)	<0.001
Age groups, years				
15−24	0.972 (0.951, 0.994)	0.013	−281 (−306, −256)	<0.001
25−34	1.076 (1.066, 1.086)	<0.001	112 (90, 133)	<0.001
35−44	1.008 (1.000, 1.015)	0.040	−17 (−37, 4)	0.101
45−54	0.999 (0.993, 1.006)	0.804	−26 (−48, −4)	0.017
55−64	0.997 (0.990, 1.004)	0.455	−18 (−47, 10)	0.202
Subgroups of depression patients, by severity				
Major vs. minor depression	1.007 (1.000, 1.013)	0.046	5 (−17, 26)	0.667
Hospitalized vs. non-hospitalized	1.013 (0.987, 1.040)	0.334	−41 (−122, 40)	0.320

CI, confidence interval

Two-by-two comparisons using *Z* tests

Employment status: age 15–24 vs. age 25–34 (*p* < 0.001); age 15–24 vs. age 35–44 (*p* = 0.001); age 15–24 vs. age 45–54 (*p* = 0.001); age 15–24 vs. age 55–64 (*p* = 0.024); age 25–34 vs. age 35–44 (*p* < 0.001); age 25–34 vs. age 45–54 (*p* < 0.001); age 25–34 vs. age 55–64 (*p* < 0.001); and age 35–44 vs. age 55–64 (*p* = 0.045); other comparisons were not significant.

Annual income: age 15–24 vs. age 25–34 (*p* < 0.001); age 15–24 vs. age 35–44 (*p* < 0.001); age 15–24 vs. age 45–54 (*p* < 0.001); age 15–24 vs. age 55–64 (*p* < 0.001); age 25–34 vs. age 35–44 (*p* < 0.001); age 25–34 vs. age 45–54 (*p* < 0.001); and age 25–34 vs. age 55–64 (*p* < 0.001); other comparisons were not significant.

Similar patterns in annual incomes were noted. The decreases in the change in trend in both genders were significant; there was no overt gender difference. In addition, the decrease in the change in trend among those aged 15–24 years was the greatest, which indicated that the gap widened the most markedly compared to other age groups. In contrast, the gap in annual income between the two groups was reduced slightly among patients aged 25–34 years. There was no significant difference in trend change among those aged 35–44 and 55–64 years. The Supplementary Figures S2 and S3 illustrated the level and trend changes in subgroup analyses.

Among patients with major depression, there was an increasing change in trend in the employment rate compared with those with minor depression after the index year; however, no difference in trend change in annual income was noted. No significant trend change in both outcomes was noted between those with or without hospitalization.

## Discussion

### Main findings

In this matched cohort study using controlled interrupted time series analysis, we found that the employment rate and annual income in the depression group were lower than those in the comparison group before the index year. Newly diagnosed depressive disorders were associated with a further decrease in the employment rate and annual income during the index year and a decreasing trend in the following years. In terms of subgroup analysis, we found that men lost more employment and annual income in the index year, but the gender difference in the change in the trend after the index year was not significant. Regarding age groups, those aged 55–64 had the most decreased employment status and annual income during the index year but did not significantly decline afterwards. In contrast, for those aged 15–24 years, the employment rate and income decreased slightly surrounding the index year; however, its difference in trend change in the following years was the most marked compared with other age groups. The severity of depression could overtly affect the patient’s employment status and annual income surrounding the index year; however, the difference in the trend was relatively small in the subsequent years.

Of note, we found that the overall employment rate and annual income kept rising after the index year in both groups. It might be due to economic growth and inflation rate in Taiwan (the GDP per capita in Taiwan increased from 31,221 USD in 2006 to 53,476 USD in 2019, an average of 4.2% growth per year; the consumer price index also increased from 84.94 in January 2006 to 100 in 98.41 in December 2019, an average of 1.07% inflation per year) (National Statistics in Taiwan). In addition, the increase was determined by the age distribution of the study sample. Supplementary Figure 3 shows that trajectories of employment rate and annual income increased markedly in young generations; however, those declined in individuals aged 55–64 years because they might retire prematurely.

### Comparison with other studies

The labour force in Taiwan is highly educated, with roughly 45% of individuals aged 25–64 years holding a bachelor’s degree or higher. This emphasis on education leads to young adults staying in school instead of entering the workforce. Additionally, the labour force is divided into 27% in manufacturing and 60% in the service sector (Ministry of Labour, 2023), with the latter being relatively lower compared to other Europe and North American countries. Despite the difference, our study is in line with previous studies, which showed that depressive disorder was associated with subsequent unemployment or low income (Andreeva *et al.*, 2015; Dobson *et al.*, 2021; Dooley *et al.*, 2000; Hakulinen *et al.*, 2021; Jefferis *et al.*, 2011; Lallukka *et al.*, 2019; Luo *et al.*, 2010; Whooley *et al.*, 2002). Our study’s trajectories of employment rate and income were compatible with longitudinal studies conducted in Canada and Finland (Dobson *et al.*, 2021; Hakulinen *et al.*, 2021). The low employment rate and income before the index year among the depression group found in our study also supported studies that showed that the association between depression and employment outcomes was bidirectional (Dobson *et al.*, 2021; Dooley *et al.*, 2000; Jefferis *et al.*, 2011). The immediate and prolonged effect of depression on employment outcomes could be attributed to the nature of the disease, which tends to relapse repeatedly and requires a longer time to recover (Hardeveld *et al.*, 2010), although some patients might only experience one depressive episode.

### Differences in the effects of depression between the genders

Compared with women, men with depressive disorders had a more obvious reduction in employment rate and income. This finding is consistent with a study conducted in Finland, which found that the likelihood of subsequent unemployment in men is higher than that in women after sick absence due to depression (Hakulinen *et al.*, 2021). Another study conducted in the United States also demonstrated that men with depressive disorders had a higher probability of being unemployed and out of the labour force than women (Luo *et al.*, 2010). The differences in treatment-seeking behaviour and social stigmatization between genders could partially explain the observed association between depression and subsequent employment outcomes. Previous studies have shown that men are likely to hesitate more to receive psychological help than women (Chang, 2007). A long duration of untreated depression may lead to poor treatment response rates and disability (Ghio *et al.*, 2015); therefore, men suffer from the adverse impact of depression on employment outcomes. In addition, women were more likely to modify their work arrangements by reducing their working hours and the pace of returning to work (De Rijk *et al.*, 2009). However, gender differences were not universal. One Swedish study showed that only women with major depression had an increased risk of unemployment compared with men (Andreeva *et al.*, 2015). The role of gender in the association between depression and employment warrants further investigation.

### Different effects of depression across working age groups

The association between depression and subsequent employment outcomes varies across age groups. The youngest age group (15–24 years) had a lower employment rate and income reduction than the older age groups. However, the impact of depression seems to backfire on employment outcomes after the year of the diagnosis. This finding might be due to most people aged 15–24 years remaining in the education system, and the overall labour force participation rate is generally low, around 29-36%, in Taiwan (Ministry of Labour, 2022). However, since adolescent-onset depression could lead to lower educational attainment (Wickersham *et al.*, 2021) and recurrent depression into adulthood (Carballo *et al.*, 2011), the youngest workforce might bear the highest risk of depression-related unemployment and low income throughout their careers. Two studies showed no association between adolescent depression and subsequent income (Callander, 2016; Naicker *et al.*, 2013); however, the null finding might be due to the small sample size.

Conversely, we found that the adults aged 45–64 years in this study were most affected by depression surrounding the index year, but the effect was not significant afterwards. Since career development tends to be stable in later life (Nagy *et al.*, 2019), the impact of depression on the employment outcomes of this age was immediate, but the long-term effect was not observed.

The raise in the employment rate in adults patients aged 25–44 and the increasing income in those aged 25–34 after the year of diagnosis might be due to adults in these age groups being traditionally seen as the prime groups of the labour force and have a relatively higher labour force participation rate, around 60–89% in Taiwan (Ministry of Labour, 2021). Therefore, they might have more opportunities to enter the labour force after temporary unemployment.

### The effect of severity of depression

We found that severity had a significant effect on employment outcomes during the year of diagnosis but not on the trend in the subsequent year. Patients with dysthymic disorders might have less symptom severity initially; however, they have a high risk of relapse and a chronic course (Klein *et al.*, 2000), adversely affecting employment outcomes. Therefore, depression severity was not significantly associated with the trend after the index year. Psychiatric hospitalization is another indicator of severity (Zimmerman *et al.*, 2018). The associations of psychiatric hospitalization with employment outcomes during the year of diagnosis are more significant than the associations of symptom severity. However, there is still no association between psychiatric hospitalization and the trend afterwards. The duration of the depressive episodes might be a stronger predictor of long-term employment outcomes than symptom severity (Lagerveld *et al.*, 2010). Our results align with prior research that demonstrated that the severity of depression at baseline did not predict subsequent low-term work disability (Rytsälä *et al.*, 2007; Sorvaniemi *et al.*, 2003).

### Limitations

This study has several limitations. First, we did not adjust the inflation rate, which was an average of 1.07% per year during the study period, and resulted in over-estimating the income. On the other hand, this study only evaluated the monthly insurance salary, which was a regular wage. Supplemental wages were not included, although generally smaller than the regular income. Overall, we thought the annual income and the difference between the two study groups were underestimated. Second, we defined employed or unemployed using an arbitrary cutoff point of 6 months. The status of partial employment was not fully considered. Furthermore, depressive disorders might change job type or employment level (full-time or part-time). Unfortunately, these variables were not available in our claims database. The income is different across job types or levels; therefore, the annual income might partially reflect the impact on job types or levels. Third, we identified newly diagnosed depressive disorders using 5-year insurance claim records to exclude prevalent cases; however, some individuals who had remote depressive episodes 6 or more years ago were not excluded. Compared to incident cases, these prevalent patients were more likely to be unemployed or have low income before the index date; however, they might have smaller changes in employment outcomes after the index year. Fourth, individuals with depressive disorders might have other comorbid mental disorders, such as anxiety disorders or sleep disorders. Because anxiety symptoms or sleep disturbance might be the prodrome or symptoms of depressive disorders, we did not exclude or adjust these comorbid conditions to avoid selection bias. The poor employment outcomes were not only attributed to depressive disorders but also the comorbid mental disorders. Therefore, the association of depression with employment outcomes might be overestimated. Moreover, perinatal depression was not identified for our analysis. Thus, the association of perinatal depression with employment outcomes is unknown. Fifth, subjects with untreated depressive disorders were misclassified into comparison groups. Thus, the impact of depression on employment may have been underestimated. Finally, several confounders, such as household size, marital status, education level, and socioeconomic status, were not measured. These factors may have influenced or modified the findings, thereby contributing to an overestimation or underestimation of our results. Despite these limitations, this study is the first to use a nationwide representative sample and longitudinal design in Asia. A controlled time series analysis was used to eliminate the effects of the time trend. The modifying effect of age on the association between depression and employment outcomes is novel.

### Healthcare policy implication

To mitigate the impact of depression on employment outcomes, healthcare policies that prioritize accessibility to mental health services and workplace accommodations may prove advantageous for individuals with depression, particularly those at heightened risk of adverse employment consequences. Additionally, policymakers could prioritize efforts to improve mental health literacy, reduce the stigmatization of mental health, and incorporate depression screening into occupational health assessments to encourage early diagnosis and treatment. Furthermore, given the significant long-term impact of depressive disorder among the youngest age group following diagnosis, support networks should be extended to encompass school systems and vocational training programs to facilitate their successful transition into the labour market. Considering premature retirement due to depression might negatively impact financial security and well-being among patients aged 55–64 years, policies could include targeted retraining programs, work accommodations, flexible work arrangements, and social welfare policies that provide income support to those who are unable to work.

## Conclusion

The present study revealed the associations between depressive disorder and poor employment outcomes, which persisted beyond the initial year of diagnosis. These findings suggest that policymakers should prioritize fortifying medical and social care for patients with depressive disorders to prevent relapse and facilitate re-entry into the labour market. Such efforts could help to narrow the employment gap between individuals with and without depressive disorders. Furthermore, our study revealed that the impact of depressive disorders on employment outcomes varied by gender and age, highlighting the necessity for further research to elucidate the potential mediating role of social context in this association. Specifically, future investigations should emphasize the development of targeted strategies to enhance employment outcomes for specific gender and age groups affected by depressive disorders.

## Data Availability

The NHIRD used in this study is held by the Taiwan Ministry of Health and Welfare. Any researcher interested in accessing the dataset can submit an application form to the Ministry of Health and Welfare requesting access, and we are not permitted to share the data.

## Supplementary material

For supplementary material accompanying this paper visit http://doi.org/10.1017/S2045796023000562. click here to view supplementary material
