# Supplementary material for: Changes in employment status and income before and after newly diagnosed depressive disorders in Taiwan: a matched cohort study using controlled interrupted time series analysis
Source: Epidemiol Psychiatr Sci. 2023 Jun 30;32:e41. doi: 10.1017/S2045796023000562 (PMC10387449; doi:10.1017/S2045796023000562)
Supplement: Supplementary file 1 [file epssup.zip › S2045796023000562sup001.docx]

**Short title:** Baseline characteristics by severity of depression

**Supplementary Table S1.** Baseline characteristics of patients with major and minor depression

|  | Before Matches | |  | After Matched | |  |
| --- | --- | --- | --- | --- | --- | --- |
|  | Major depression, N (%) | Minor depression, N (%) | STDDIF | Major Depression, N (%) | Minor Depression, N (%) | STDDIF |
| **Age Category** |  |  |  |  |  |  |
| 15-24 | 35 933 (10.9) | 11 052 (12.1) | -0.04 | 11 052 (12.1) | 11 052 (12.1) | 0.00 |
| 25-34 | 61 830 (18.8) | 18 296 (20.0) | -0.03 | 18 296 (20.0) | 18 296 (20.0) | 0.00 |
| 35-44 | 72 913 (22.1) | 20 414 (22.3) | 0.00 | 20 414 (22.3) | 20 414 (22.3) | 0.00 |
| 45-54 | 82 458 (25.0) | 22 464 (24.5) | 0.01 | 22 464 (24.5) | 22 464 (24.5) | 0.00 |
| 55-64 | 76 182 (23.1) | 19 393 (21.2) | 0.05 | 19 393 (21.2) | 19 393 (21.2) | 0.00 |
| **Gender** |  |  |  |  |  |  |
| Men | 125 930 (38.2) | 32 006 (34.9) | 0.07 | 32 006 (34.9) | 32 006 (34.9) | 0.00 |
| Women | 203 386 (61.8) | 59 613 (65.1) | -0.07 | 59 613 (65.1) | 59 613 (65.1) | 0.00 |
| **Index year** |  |  |  |  |  |  |
| 2011 | 86 172 (26.2) | 25 008 (27.3) | -0.03 | 25 008 (27.3) | 25 008 (27.3) | 0.00 |
| 2012 | 83 190 (25.3) | 23 524 (25.7) | -0.01 | 23 523 (25.7) | 23 524 (25.7) | 0.00 |
| 2013 | 80 538 (24.5) | 21 798 (23.8) | 0.02 | 21 798 (23.8) | 21 798 (23.8) | 0.00 |
| 2014 | 79 416 (24.1) | 21 289 (23.2) | 0.02 | 21 290 (23.2) | 21 289 (23.2) | 0.00 |
| **Residential area** |  |  |  |  |  |  |
| Urban | 172 530 (52.4) | 49 282 (53.8) | -0.03 | 49 282 (53.8) | 49 282 (53.8) | 0.00 |
| Suburban | 122 913 (37.3) | 32 918 (35.9) | 0.03 | 32 921 (35.9) | 32 918 (35.9) | 0.00 |
| Rural | 33 873 (10.3) | 9419 (10.3) | 0.00 | 9416 (10.3) | 9419 (10.3) | 0.00 |
| **CCI score** |  |  |  |  |  |  |
| 0 | 234 478 (71.2) | 64 970 (70.9) | 0.01 | 64 972 (70.9) | 64 970 (70.9) | 0.00 |
| 1' | 53 983 (16.4) | 14 757 (16.1) | 0.01 | 14 757 (16.1) | 14 757 (16.1) | 0.00 |
| 2' | 21 580 (6.6) | 6077 (6.6) | 0.00 | 6076 (6.6) | 6077 (6.6) | 0.00 |
| 3 or more | 19 275 (5.9) | 5815 (6.3) | -0.02 | 5814 (6.3) | 5815 (6.3) | 0.00 |
| STDDIF standardised difference; Index Year= Year of depression diagnosis; CCI Charlson comorbidity index | | | | | | |

**Short title:** Hospitalized and non-hospitalized patients’ baseline characteristics

**Supplementary Table S2.** Baseline characteristics of hospitalized and non-hospitalized patients with depression

|  | Before Matches | |  | After Matched | |  |
| --- | --- | --- | --- | --- | --- | --- |
|  | Non-hospitalised depression, N (%) | Hospitalised depression, N (%) | STDDIF | Non-hospitalised depression, N (%) | Hospitalised depression, N (%) | STDDIF |
| **Age Category** |  |  |  |  |  |  |
| 15-24 | 46 006 (11.1) | 979 (17.2) | -0.18 | 979 (17.2) | 979 (17.2) | 0.00 |
| 25-34 | 78 994 (19.0) | 1132 (19.9) | -0.02 | 1132 (19.9) | 1132 (19.9) | 0.00 |
| 35-44 | 91 981 (22.2) | 1346 (23.7) | -0.04 | 1346 (23.7) | 1346 (23.7) | 0.00 |
| 45-54 | 103 607 (25.0) | 1,315 (23.1) | 0.04 | 1315 (23.1) | 1315 (23.1) | 0.00 |
| 55-64 | 94 662 (22.8) | 913 (16.1) | 0.17 | 913 (16.1) | 913 (16.1) | 0.00 |
| **Gender** |  |  |  |  |  |  |
| Men | 154 848 (37.3) | 3088 (54.3) | -0.35 | 3088 (54.3) | 3088 (54.3) | 0.00 |
| Women | 260 402 (62.7) | 2597 (45.7) | 0.35 | 2597 (45.7) | 2597 (45.7) | 0.00 |
| **Index year** |  |  |  |  |  |  |
| 2011 | 109 646 (26.4) | 1534 (27.0) | -0.01 | 1534 (27.0) | 1534 (27.0) | 0.00 |
| 2012 | 105 267 (25.4) | 1447 (25.5) | 0.00 | 1447 (25.5) | 1447 (25.5) | 0.00 |
| 2013 | 101 026 (24.3) | 1310 (23.0) | 0.03 | 1310 (23.0) | 1310 (23.0) | 0.00 |
| 2014 | 99 311 (23.9) | 1394 (24.5) | -0.01 | 1394 (24.5) | 1394 (24.5) | 0.00 |
| **Residential area** |  |  |  |  |  |  |
| Urban | 219 012 (52.7) | 2800 (49.3) | 0.07 | 2800 (49.3) | 2800 (49.3) | 0.00 |
| Suburban | 153 703 (37.0) | 2128 (37.4) | -0.01 | 2128 (37.4) | 2128 (37.4) | 0.00 |
| Rural | 42 535 (10.2) | 757 (13.3) | -0.10 | 757 (13.3) | 757 (13.3) | 0.00 |
| **CCI score** |  |  |  |  |  |  |
| 0 | 295 527 (71.2) | 3921 (69.0) | 0.05 | 3921 (69.0) | 3921 (69.0) | 0.00 |
| 1' | 67 785 (16.3) | 955 (16.8) | -0.01 | 955 (16.8) | 955 (16.8) | 0.00 |
| 2' | 27 263 (6.6) | 394 (6.9) | -0.01 | 394 (6.9) | 394 (6.9) | 0.00 |
| 3 or more | 24 675 (5.9) | 415 (7.3) | -0.05 | 415 (7.3) | 415 (7.3) | 0.00 |
| STDDIF standardised difference; Index Year= Year of depression diagnosis; CCI Charlson comorbidity index | | | | | | |

**Supplementary Table 3.** The employment rate, annual income, and difference between case and control groups.

|  | Employment rate (%) | | | Annual Income (USD) | | | | |
| --- | --- | --- | --- | --- | --- | --- | --- | --- |
| Year | Case | Control | Difference | Case | Control | Difference |  |  |
| -5 | 60.7 | 66.0 | 5.2 (5.0, 5.5) | 7113 | 8091 | 977 (974, 981) |  |  |
| -4 | 61.0 | 66.3 | 5.3 (5.1, 5.5) | 7285 | 8298 | 1013 (1010, 1016) |  |  |
| -3 | 61.5 | 66.8 | 5.3 (5.1, 5.5) | 7482 | 8524 | 1042 (1042, 1045) |  |  |
| -2 | 61.9 | 67.2 | 5.3 (5.1, 5.6) | 7660 | 8738 | 1078 (1074, 1081) |  |  |
| -1 | 61.9 | 67.6 | 5.7 (5.5, 5.9) | 7812 | 8987 | 1172 (1172, 1175) |  |  |
| 0 | 61.1 | 67.8 | 6.7 (6.5, 7.0) | 7838 | 9217 | 1379 (1379, 1382) |  |  |
| 1 | 61.6 | 68.8 | 7.3 (7.0, 7.5) | 7981 | 9553 | 1573 (1570, 1576) |  |  |
| 2 | 62.3 | 69.7 | 7.4 (7.2, 7.6) | 8184 | 9864 | 1683 (1680, 1683) |  |  |
| 3 | 63.1 | 70.7 | 7.6 (7.4, 7.8) | 8434 | 10217 | 1783 (1780, 1786) |  |  |
| 4 | 63.9 | 71.8 | 7.9 (7.6, 8.1) | 8696 | 10592 | 1896 (1893, 1900) |  |  |
| 5 | 64.6 | 72.7 | 8.1 (7.9, 8.4) | 8958 | 10964 | 2006 (2003, 2010) |  |  |

**Supplementary Table 4.** Overall controlled interrupted time-series analysis, using sample before propensity-score matching with adjustment for Charlson comorbid index score

|  | **Employment status** |  | **Annual income** |  |
| --- | --- | --- | --- | --- |
| **Variables** | **Odds ratios (95% CI)** | **p-value** | **Coefficient (95% CI)** | **p-value** |
| Intercept (β0) | 2.106 (2.095, 2.117) | <.001 | 8924 (8904, 8944) | <.001 |
| Year (β1) | 1.034 (1.033, 1.035) | <.001 | 274 (270, 278) | <.001 |
| Phase (β2) | 1.013 (1.009, 1.017) | <.001 | 17 (6, 28) | 0.003 |
| Phase*Year (β3) | 0.997 (0.996, 0.999) | 0.002 | -9 (-14, -4) | 0.001 |
| Depression (β4) | 0.774 (0.768, 0.779) | <.001 | -1125 (-1151, -1098) | <.001 |
| Depression*Year (β5) | 0.993 (0.992, 0.995) | <.001 | -26 (-32, -21) | <.001 |
| Depression*Phase (β6) | 0.963 (0.958, 0.969) | <.001 | -225 (-241, -209) | <.001 |
| Depression*Phase*Year (β7) | 0.997 (0.995, 0.999) | 0.011 | -48 (-55, -40) | <.001 |

CI confidence interval; β1. Trends in the control groups; β2. Level Change in the Index Year for the Control Group; β3. Trend change after the index year for the control groups; β4. Difference in intercept in index year between the case and control groups; β5. Trend differences between case and control groups; β6. Difference in level change in the index year between the case and control groups; β7. Difference in trend change after the index year between the case and control groups

Supplementary Figure 1. Timeframe of study design

Supplementary Figure 2. Employment rate and annual income in case and control groups before and after the index year, by genders

Supplementary Figure 3. Employment rate and annual income in case and control groups before and after the index year, by age groups
