# Supplementary figures and images for: Changes in employment status and income before and after newly diagnosed depressive disorders in Taiwan: a matched cohort study using controlled interrupted time series analysis
Source: Epidemiol Psychiatr Sci. 2023 Jun 30;32:e41. doi: 10.1017/S2045796023000562 (PMC10387449; doi:10.1017/S2045796023000562)

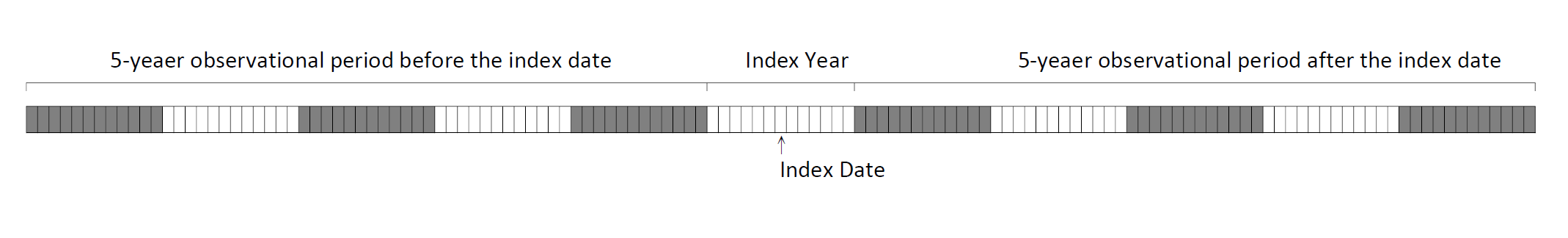

Supplement: Supplementary file 1 [file epssup.zip › S2045796023000562sup002.png]

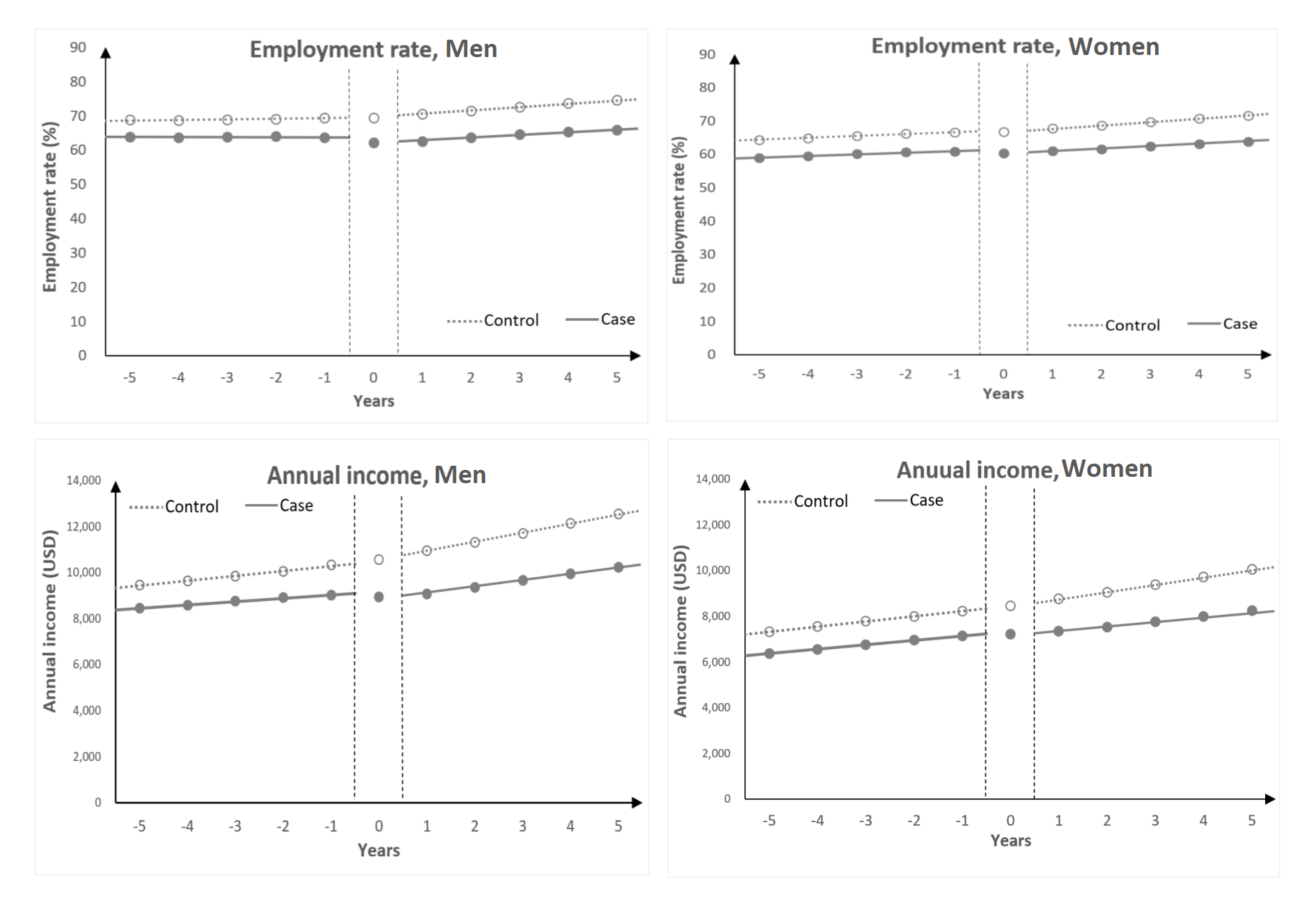

Supplement: Supplementary file 1 [file epssup.zip › S2045796023000562sup003.tif]

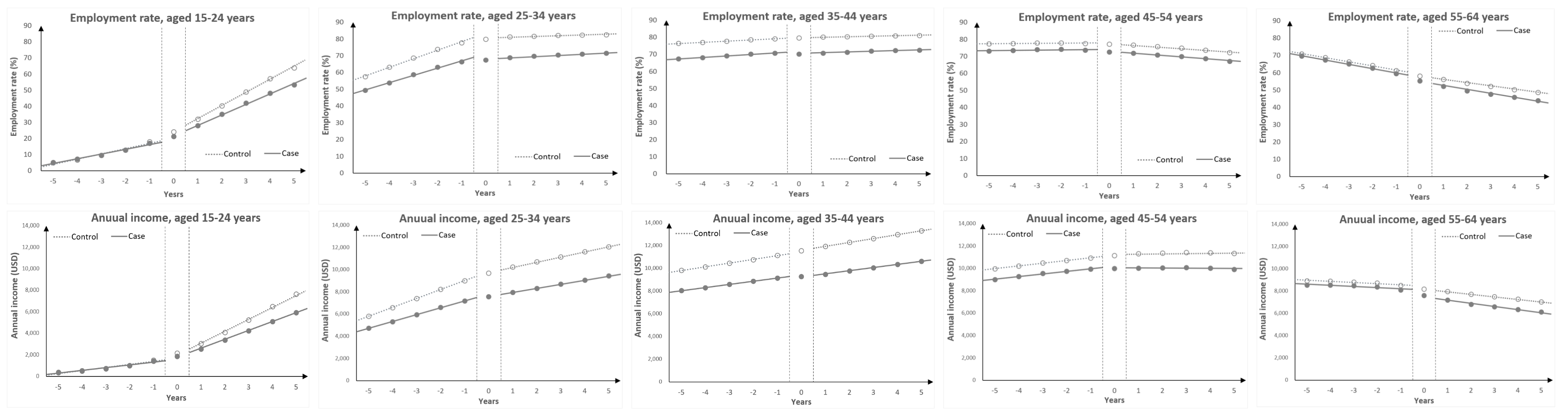

Supplement: Supplementary file 1 [file epssup.zip › S2045796023000562sup004.tif]
